# New Infectious Diseases and Industrial Food Animal Production

**DOI:** 10.3201/eid1609.100144

**Published:** 2010-09

**Authors:** Ellen Silbergeld, Meghan Davis, Bath Feingold, Alan Goldberg, Jay Graham, Jessica Leibler, Amy Peterson, Lance B. Price

**Affiliations:** Author affiliations: Johns Hopkins School of Public Health, Baltimore, Maryland, USA (E. Silbergeld, M. Davis, B. Feingold, A. Goldberg, J. Leibler, A. Peterson);; American Association for the Advancement of Science, Washington, DC, USA (J. Graham);; Center for Metagenomics and Human Health Translational Genomics Research Institute, Flagstaff, Arizona, USA (L.B. Price)

**Keywords:** Antimicrobial resistance, agriculture, MRSA, methicillin-resistant Staphylococcus aureus, zoonoses, bacteria, letter

**To the Editor**: Cutler et al. bring welcome attention to the importance of new and reemerging zoonotic diseases in the industrialized world (*1*). However, they make no mention of industrialized systems of food animal production, major sources of antimicrobial drug–resistant bacterial pathogens (*2*) that are among the most globally prevalent and emerging infectious diseases (*3*). These systems have practices characterized by crowded and unsanitary confinement of animals and routine use of antimicrobial agents in animal feeds (*2*). For example, in the same issue, Dutil et al. (*3*) reported on increases in ceftiofur resistance in *Salmonella enterica* isolates from food, which they associate with use of this drug in broiler poultry production.

Recognition of the role of industrial food animal production in driving vancomycin resistance in enterococci prompted restrictions on agricultural antimicrobial drug use in the European Union; unfortunately, few measures have been implemented in the rest of the world (including the United States) (*4*). Industrialized food animal production is now assumed to contribute to the emergence of new strains of community-associated methicillin-resistant *Staphylococcus aureus* with varying potential for infecting humans (*5*). Because the industrial model of food animal production is rapidly expanding globally (*2*), this source must be included in surveillance, research, and tracking programs for effective prevention of emerging zoonotic disease.
