# Correction—Vol. 16, No. 9

**DOI:** 10.3201/eid1612.C21612

**Published:** 2010-12

**Keywords:** errata, erratum, Correction, Vol. 16, No. 9

The name of author Beth Feingold was misspelled in the letter New Infectious Diseases and Industrial Food Animal Production (E. Silbergeld et al.). The article has been corrected online (http://wwwnc.cdc.gov/eid/article/16/9/10-0144_article.htm).

