# Supplementary material for: New Infectious Diseases and Industrial Food Animal Production
Source: Emerg Infect Dis. 2010 Sep;16(9):1503–4. doi: 10.3201/eid1609.100144 (PMC3298288; doi:10.3201/eid1609.100144)
Supplement: Related texts

# Reply: New Infectious Diseases and Industrial Food Animal Production

**Authors:** Sally J. Cutler, Anthony R. Fooks, Wim H.M. van der Poel

**Affiliations:** Author affiliations: University of East London, London, UK (S.J. Cutler);; University of Liverpool, Liverpool, UK (A.R. Fooks);; Veterinary Laboratories Agency, Weybridge, UK (A.R. Fooks);; Central Veterinary Institute, Lelystad, the Netherlands (W.H.M. van der Poel)

**In Response:** Silbergeld et al. highlight pertinent points about how stochastic events can lead organisms to acquire adaptive advantages through lateral gene transfer (*1*). Word constraints of our earlier article precluded detailed debate of many such topics, and we welcome the opportunity to discuss this further. The role of industrial food animal production in driving the development of antimicrobial drug–resistant pathogens is indeed a topic of great concern.

Commonly, reemergence of infections is caused by changes in the environment or the host, genetic changes of pathogens, or alteration in the dynamic interactions that unite them. Our need for intensive protein production can have explosive consequences, as seen with the recent outbreak of Q fever among humans residing near goat farming areas in the Netherlands (*2*) and the emergence of antimicrobial drug–resistant organism variants with selective advantages, such as methicillin-resistant *Staphylococcus aureus* (*3*). The bombardment of livestock with antimicrobial drugs for therapy and prophylaxis and as growth-enhancing agents (in Europe before 2006) has provided selective pressure for acquisition of resistance, which occurs globally (*4*). Even exposure to various biocides has been linked with acquisition of resistance to therapeutic antimicrobial agents (*5*), although such resistance has not yet been demonstrated in natural populations. Risk prevention within and management of intensified food production systems is a continuing challenge. Similarly problematic are pathogens that increase in general, such as RNA viruses that under the recent selective pressure have rapidly acquired resistance to oseltamivir (*6*). A common feature of all these facts is that such traits and clones of increased fitness can disseminate rapidly around the globe. For these reasons, we need robust surveillance mechanisms; ability to predict spread; cohesive intervention strategies; and lastly, but by no means least, strong collaborative links between previously segregated human and veterinary fields that extend to producers and policy makers.

## References

[1] Silbergeld E, David M, Feingold B, Goldberg A, Graham J, Leibler J, New infectious diseases and industrial food animal production. Emerg Infect Dis. 2010;16:1503.2073595110.3201/eid1609.100144PMC3298288

[2] Karagiannis I, Schimmer B, Van Lier A, Timen A, Schneeberger P, Van Rotterdam B, Investigation of a Q fever outbreak in a rural area of the Netherlands. Epidemiol Infect. 2009;137:1283–94. 10.1017/S095026880800190819161644

[3] van Loo I, Huijsdens X, Tiemersma E, de Neeling A, van de Sande-Bruinsma N, Beaujean D, Emergence of methicillin-resistant *Staphylococcus aureus* of animal origin in humans. Emerg Infect Dis. 2007;13:1834–9.1825803210.3201/eid1312.070384PMC2876750

[4] Hawkey PM, Jones A. The changing epidemiology of resistance. J Antimicrob Chemother. 2009;64(Suppl 1):i3–10. 10.1093/jac/dkp25619675017

[5] Maseda H, Hashida Y, Konaka R, Shirai A, Kourai H. Mutational upregulation of a resistance-nodulation-cell division–type multidrug efflux pump, SdeAB, upon exposure to a biocide, cetylpyridinium chloride, and antibiotic resistance in *Serratia marcescens.* Antimicrob Agents Chemother. 2009;53:5230–5. 10.1128/AAC.00631-0919752278PMC2786342

[6] Sy CL, Lee SS, Liu MT, Tsai HC, Chen YS. Rapid emergence of oseltamivir resistance. Emerg Infect Dis. 2010;16:723–5.2035040210.3201/eid1604.091706PMC3321971
